# Relationship of excessive daytime sleepiness with bruxism, depression, anxiety, stress, and sex in odontology students – A cross sectional study

**DOI:** 10.4317/jced.59049

**Published:** 2022-06-01

**Authors:** Karina-Helga Turcio, Clóvis-Lamartine de Moraes-Melo-Neto, Fernanda-Pereira de Caxias, Marcelo-Coelho Goiato, Daniela-Micheline dos Santos, Marcella-Santos Januzzi, Aimée-Maria Guiotti, André-Pinheiro-de Magalhães Bertoz, Daniela-Atili Brandini

**Affiliations:** 1 DDS, MS, PhD. Department of Dental Materials and Prosthodontics, Araçatuba Dental School, São Paulo State University (UNESP), Araçatuba, São Paulo, Brasil; 2 DDS, MS, PhD. Oral Oncology Center, São Paulo State University (UNESP), School of Dentistry, Araçatuba, São Paulo, Brazil; 3 DDS, MS. Department of Dental Materials and Prosthodontics, Araçatuba Dental School, São Paulo State University (UNESP), Araçatuba, São Paulo, Brasil; 4 DDS, MS, PhD. Department of Pediatric and Social Dentistry, São Paulo State University (UNESP), School of Dentistry, Araçatuba, São Paulo, Brazil; 5 DDS, MS, PhD. Department of Diagnosis and Surgery, Araçatuba Dental School, São Paulo State University (UNESP), São Paulo, Brasil

## Abstract

**Background:**

To verify if there is a correlation of excessive daytime sleepiness (EDS) with bruxism, depression, anxiety, stress, and sex in odontology students.

**Material and Methods:**

Four hundred dental students were selected. Students included in the study were those that were healthy, over 18 years old, and with similar weekly academic activities. Students excluded were those with temporomandibular disfunction, a high possibility of possessing obstructive sleep apnea and illnesses that cause EDS; along with smokers, users of illicit drugs, users of psychiatric medication, and those that abuse the consumption of alcohol. After the application of these criteria, 128 students were included in this study. EDS, bruxism, depression, anxiety, and stress were evaluated by the Epworth Sleepiness Scale, the Pintado et al. questionnaire, Beck Depression Inventory, Beck Anxiety Inventory, and the LIPP Adult Stress Symptom Inventory. Afterwards, the Spearman test (*p*< 0.05) was applied.

**Results:**

A high probability of EDS was present in 33.6% of the students. There was a positive correlation of EDS with females (Rank Correlation= 0.209; *p*= 0.018) and depression (Rank Correlation= 0.174; *p*= 0.049); between females and stress (Rank Correlation= 0.199; *p*= 0.024), and between females and anxiety (Rank Correlation= 0.178; *p*= 0.045). There was a positive correlation of bruxism with anxiety (Rank Correlation= 0.255; *p*= 0.004) and stress (Rank Correlation= 0.201; *p*= 0.023). There was no correlation of EDS with bruxism (*p*= 0.354), stress (*p*= 0.277), or anxiety (*p*= 0.114). There was no correlation of bruxism with females (*p*= 0.082) or depression (*p*= 0.362).

**Conclusions:**

A high probability of EDS was present in 33.6% of dentistry students, and there was a positive correlation of EDS with females and depression.

** Key words:**Bruxism, depression, anxiety, stress, disorders of excessive somnolence, dentistry.

## Introduction

Healthy sleep is an important physiologic phenomenon (1) for good health in general (2) and the following: learning, reasoning, mental and physical restoration (1), concentration, judgement (3), memory consolidation, cellular growth (4), good mood (5,6), realization of daily activities with efficiency (6), functioning of the immunologic system, state of alertness and adequate reaction time, good quality of life (3), and security in transit and work (7). It depends on factors that include an adequate duration, good quality, and an absence of sleep disturbances (2). Therefore, an unhealthy sleep could prejudice all of the initially cited situations.

The knowledge obtained in a university is of grand importance in providing emerging students with abilities and independence to plot their own way, to have a successful job, and to contribute to society (5). The obstacles to maximize the success of college students include irregular hours of sleep, sleep deprivation, and daytime sleepiness (5). Thus, healthy sleep is very important, principally for odontology students, since odontology colleges explore arduous clinical and theoretic content, require long hours of study and practice, and high levels of concentration and dexterity (6).

Excessive daytime sleepiness (EDS) is defined as a difficulty to remain awake and alert during the day (5,8,9). Individuals with EDS could sleep involuntarily or non-intentionally during the execution of their daily activities (5,8). EDS could be a sign of insufficient sleep, inadequate sleep habits, and even illness, such as respiratory disturbances associated to sleep, restless legs syndrome, circadian rhythm disturbances, chronic obstructive pulmonary disease, stroke, asthma, and narcolepsy (9). In addition, obesity could be related to EDS (9). Therefore, it is extremely important to discover what factors could cause and are related to EDS in university students, so that the most rapid diagnosis, treatment, and help to avoid the “problem” affecting other students can be performed.

Articles in the literature have shown that odontology students have problems in relation to sleep (1,10-15). In 2014, in evaluating Brazilian odontology students, it was verified that they slept 6.8 hours per night (less than recommended), and as a possible consequence, 79.2% reported difficulty of concentration in daytime activities (1). In addition, in that study, there was also a significant positive correlation of duration of sleep with sleep bruxism and daytime bruxism (both self-reported) (1). In 2016, in Saudi Arabian odontology students, a significant positive correlation of self-reported bruxism was observed at the beginning of sleep, during nighttime sleep, during daytime naps, awakening early in the morning before the usual hour without a cause, and also an increase in nightmares (10). In the same year (2016), a significant positive correlation was also observed of insomnia with addiction to the Internet, stress, anxiousness, and depression in odontology, medical, and pharmacy students (14). In 2020, upon evaluating odontology, medicine, nursing, optometry, pharmacy, social service, and veterinarian students, it was observed that 17% reported moderate to severe depressive symptoms, with 6% reporting thoughts of suicide (1); Also, 14% reported moderate to severe anxiety (11); and factors that predict depression and anxiety included less than 7 hours of sleep per night and greater stress, among others (11). In addition, other studies reported that odontology students had lack of sleep (12), poor quality of sleep, and sleepiness while performing academic tasks (13).

Sleep bruxism is included among the movement of disturbances related to sleep (1,16). It is suggested that bruxism could be caused by three groups of factors: Group 1 – Biologic factors (which include neurotransmitters, e.g., dopamine), cortical awakenings, and genetic factors; Group 2 – Factors of exogenous origin, which include nicotine, caffeine, alcohol, drugs, and some medication (e.g. fluoxetine); and Group 3 – Psychologic factors, which include sensitivity to stress, individual character traits, and anxiousness, among others (17). It is important to mention that odontology students present more elevated levels of depression, anxiety, and stress than the general population, indicating they could be at risk of greater psychological suffering (18). Thus, based on the information in the last paragraph, it is fundamental to discover if there is a correlation between EDS and bruxism, depression, anxiety, stress, and sex (male or female) in odontology students.

After performing a search using these combinations of keywords (Mesh terms): “students”, “sleep”, and “dentistry” with “bruxism” or “stress” or “anxiety” or “depression” or “disorders of excessive somnolence”, it was possible to verify that there were no studies evaluating EDS in dentistry students. Therefore, the objective of this study was to verify if there is a correlation of EDS with bruxism, depression, anxiety, stress, and sex in odontology students.

## Material and Methods

This transversal study was approved by the ethics committee (number: 30367314.2.0000.5420) of the Araçatuba Dental School (São Paulo State University), and was performed from September 2017 until March 2018, according to the Helsinki declaration. All participants were informed about the study and signed a free and informed consent form.

The sample size was calculated from a finite population of 400 dental students. The minimum sample required for a 90% confidence interval was 122 subjects, with a 5% margin of error. Thus, after interviewing the 400 students, 128 subjects (97 women and 31 men) were included in this study, based on the inclusion and exclusion criteria.

•Inclusion criteria

-Students (men and women) of odontology from the Araçatuba Dental School (public university).

-Healthy students.

-Above 18 years of age.

-All had to have similar weekly academic activities (practical and theoretic classes, as well as attend patients).

•Exclusion criteria

-Students with a high probability of possessing obstructive sleep apnea (OSA) (7,9), verified by the Berlin questionnaire (19).

-Smokers, since smoking could prejudice sleep (20) and induce bruxism (17).

-Abusive consumption of alcoholic beverages, since alcohol could prejudice sleep (5) and induce bruxism (17).

-Temporomandibular dysfunction, verified by the RDC (Research Diagnostic Criteria for Temporomandibular Disorders) questionnaire, since it could prejudice sleep (15).

-Those with some systemic pathology that could have a negative influence on sleep (e.g., pulmonary illnesses) (9,10).

-Use of illicit drugs, since they could have a negative influence on sleep (7,21) and induce bruxism (17).

-Use of psychiatric medication, which could induce bruxism (22) and alter sleep patterns (7,10).

-Use of medication associated with EDS (7).

-Kleine-Levin Syndrome (7).

-Cancer.

-Participants of chemotherapy or radiotherapy treatment.

-Those that were not willing to participate in the study.

•Berlin Questionnaire

The Berlin Questionnaire used in the present study was validated for the Portuguese language (Brazil) (19). This questionnaire is a screening tool used to differentiate individuals with a high or low chance of having OSA. This questionnaire consists of 10 items, divided into three categories (1. snoring and witnessed apnea, 2. daytime sleepiness, and 3. arterial hypertension/obesity). According to Andrechuk *et al*. (2019): Category 1 ranges from 0 to 6 points and is considered positive when the score is 2 points or more; Category 2 ranges from 0 to 3 points and is considered positive when the score is 2 points or more; and Category 3 will be positive if the participant reports that he/she has high blood pressure or a body mass index (BMI) >30 kg/m2 (19). The final score indicates a high risk of OSA when two or more categories are positive. A low risk of OSA is indicated when all of the categories had no positive score or when there was a positive score in only one category.

•Instructions for the participants

All included individuals were instructed to: 1) not take medication and/or alcoholic beverages prior to the application of the questionnaires; and 2) to respond to the questionnaires based on their daily academic routine, disregarding days outside of the routine.

The questionnaires used were the Epworth Sleepiness Scale (23), Pintado *et al*. (1997) questionnaire (24), Beck Depression Inventory (25), Beck Anxiety Inventory (26), and the LIPP Adult Stress Symptom Inventory (27).

•Epworth Sleepiness Scale

The students were evaluated by means of the Epworth Sleepiness Scale, validated in the Portuguese language (Brazil) (23). This is a self-report questionnaire that evaluates the probability of sleep in eight different situations. The possible answers of the questions are: “would never doze” – representing “0”; “slight chance of dozing” - representing “1”; “moderate chance of dozing” – representing “2”; and “high chance of dozing” – representing “3”. Afterwards, a summation is performed with the numbers collected from each question. The sum of points varies from 0 to 24. It is worth restating that a score of 0 – 10 represents a low possibility of EDS; and above 10 suggest a high probability of EDS.

•Pintado *et al*. questionnaire

The identification of bruxism was based on the *Pi*ntado *et al*. (1997) questionnaire, involving the following questions (24): 1. Has anyone heard you grinding your teeth at night?; 2. Is your jaw ever fatigued or sore on awakening in the morning?; 3. Are your teeth or gums ever sore on awakening in the morning?; 4. Do you ever experience temporal headaches on awakening in the morning?; 5. Are you ever aware of grinding your teeth during the day?; and 6. Are you ever aware of clenching your teeth during the day? Each participant could have responded “yes” or “no” for each question, and one positive response (“yes”) already classified the individual as having bruxism.

•Beck Depression Inventory 

The Beck Depression Scale or Beck Depression Inventory, consists of a self-report questionnaire and is an instrument for measuring the severity of depression episodes (25). It consists of 21 items that include symptoms and attitudes, with intensities ranging from neutral to a maximum level of severity, classified from 0 to 3. The sum of the scores of each question may generate the following results: 0-13 minimal depression; 14-19 mild depression; 20-28 moderate depression; and 29-63 severe depression. It is important to mention that this questionnaire was validated for the Portuguese language in Brazil (25).

•Beck Anxiety Inventory

The Beck Anxiety Scale or Beck Anxiety Inventory, also developed by Beck, is a self-report questionnaire that measures the severity of an individual’s anxiety (26). It consists of 21 questions about how the individual has felt in the past week, expressed in common anxiety symptoms (such as fear of losing control and sweating). Each question has 4 possible responses that reflect the level of severity for each symptom: 0 -“Absolutely not”; 1 - “Lightly - it did not bother me much”; 2 - “Moderately - It was very unpleasant, but I could bear it”; and 3 - “Gravely - I could hardly bear it.” The sum of the scores of each question may generate the following results: 0-10 minimal anxiety; 11-19 mild anxiety; 20-30 moderate anxiety; and 31-63 severe anxiety. It is important to mention that this questionnaire was validated for the Portuguese language in Brazil (26).

•LIPP Adult Stress Symptom Inventory 

The LIPP Adult Stress Symptom Inventory provides a measure of stress symptoms in adults and was validated for the Portuguese language (Brazil) (27). It consists of three frameworks to delineate the phases of stress, with the first referring to the physical or psychological symptoms that the person has experienced in the last 24 hours, being composed of 15 questions. The second framework refers to the symptoms experienced in the last 7 days, consisting of ten physical and 5 psychological symptoms (15 questions). The third and final frame refers to symptoms experienced in the last month, consisting of 12 physical and 11 psychological symptoms (23 questions). Some symptoms are repeated in more than one framework, but at different intensities.

The theoretical model of stress is quadriphasic, divided into the following phases: Alert Phase, Resistance Phase, Nearly Exhausted Phase, and Exhausted Phase (27). The Alert Phase is the positive phase of stress, when the individual automatically prepares for action. If the Alert Phase is maintained for a long time or if new stressors accumulate, the Resistance Phase enters, in which the organism tries to prevent the total waste of energy and the individual unconsciously tries to reestablish the inner balance. The Near Exhaustion Phase (newly discovered phase) is when tension exceeds the limit of the manageable. At that point, the physical and emotional resistance begins to break, even though there are still moments when the individual can think lucidly, albeit with much effort. There is a lot of anxiety at this stage. The Exhaustion Phase is the most negative phase of pathological stress, with a great inner imbalance. The individual goes into depression and is unable to concentrate or work. The following scores are assigned to determine the stress phase: 1 - No stress; 2 - Alert phase; 3 - Resistance phase; 4 - Near exhaustion phase; and 5 - Exhaustion phase. To determine the physical and psychological symptoms of stress, the scores are: 1 - Alert phase; 2 - Resistance / near exhaustion phase; and 3 - Exhaustion phase.

•Statistic analysis

The data collected by means of the questionnaires were tabulated. According to the responses obtained by the Epworth Sleepiness Scale, the participants were divided in 2 groups: without EDS (Group 1) and with EDS (Group 2). Then, the data were submitted to statistical analysis using the IBM SPSS 20.0 (Statistical Package for the Social Sciences, IBM, USA). The Spearman test (*p* < 0.05) was applied to verify if there was a correlation among the collected data.

## Results

The mean age of the included individuals was 21 years old. The sample percentage data were presented in Table 1. Of the 128 participants, 85 showed a low probability of having EDS, and 43 showed a high probability of having EDS.

**Table 1:**
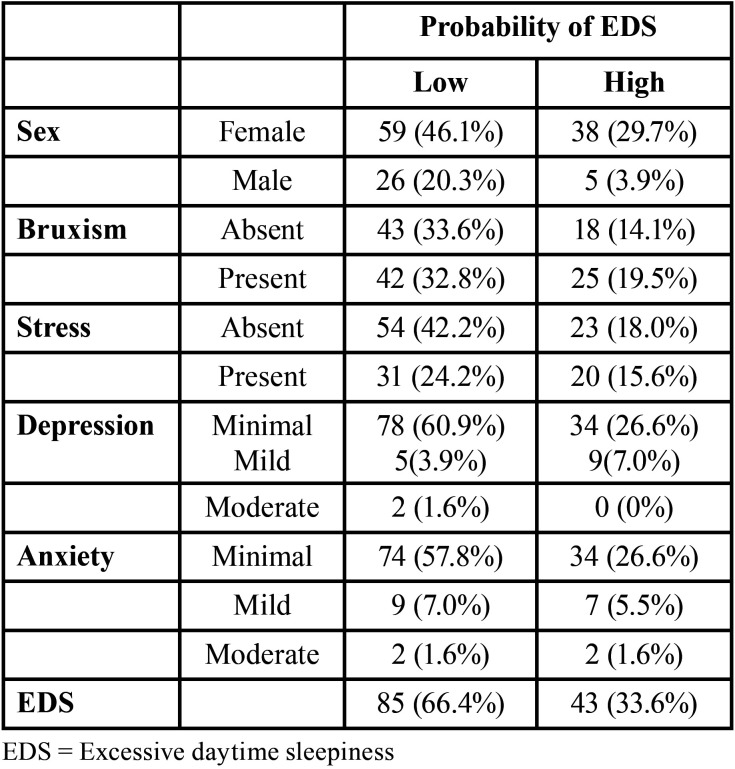
Percentage analysis of the studied sample.

Through the Pintado *et al*. questionnaire, 32.8% of the students with a low probability of having EDS and 19.5% of the students with a high probability of having EDS reported having daytime bruxism and nighttime bruxism (Table 1). Therefore, the Pintado *et al*. questionnaire identified a possible presence of both types of bruxism, but did not identify which type of bruxism was more prevalent in each individual.

Table 2 shows that there was a positive correlation between EDS and females (Rank Correlation= 0.209; *p*= 0.018); and between EDS and depression (Rank Correlation= 0.174; *p*= 0.049). There was no correlation of EDS with bruxism (*p*= 0.354), stress (*p*= 0.277), or anxiety (*p*= 0.114) (Table 2).

Bruxism did not correlate with females (*p*= 0.082) and depression (*p*= 0.362). Despite that, there was a positive correlation of bruxism with anxiety (Rank Correlation= 0.255; *p*= 0.004) and stress (Rank Correlation= 0.201; *p*= 0.023) (Table 2).

**Table 2:**
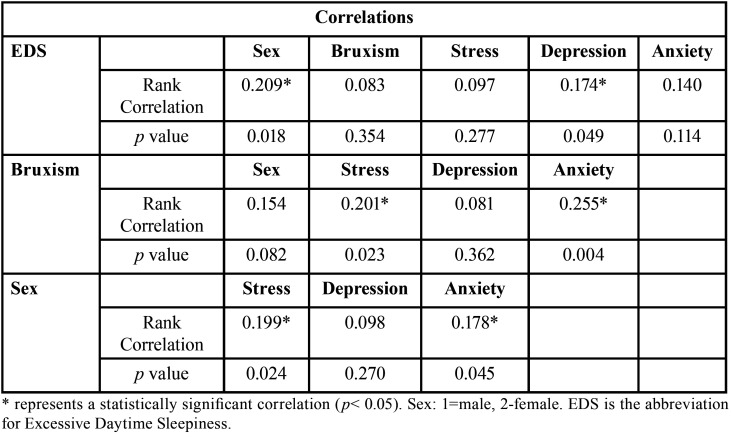
Correlations evaluated in this study.

There was a positive correlation between females and stress (Rank Correlation= 0.199; *p*= 0.024), and between females and anxiety (Rank Correlation= 0.178; *p*= 0.045) (Table 2).

## Discussion

The polysomnography of an entire night is the gold standard for the diagnosis of sleep bruxism (20), just as the multiple sleep latency test and the maintenance of wakefulness test represent the gold standard for diagnosis of EDS (8). However, these tests were not used in the present study, due to the high costs involved. Therefore, the Pintado *et al*. (1997) questionnaire (24) and the Epworth Sleepiness Scale were used (23), since they are used in studies in the literature (7,8,9,28,29).

Of the conditions that cause EDS, OSA is the most frequent diagnosis (9). In this study, participants with a high probability of having OSA were excluded, since these people could have EDS as a consequence of OSA (7,9); and thus it would not be possible to verify correctly if there would be a correlation of EDS with bruxism, anxiety, stress, depression, and sex. Thus, OSA could represent a bias. In addition, other possible biases were excluded from this study, as reported in the exclusion criteria of the methodology.

The mean age of individuals included in this study was 21 years old. Although the traditional period of adolescence is considered from 12 to 18 years old, currently, this period can be extended until the age of 25, to include the young adult age (30). Based on this result (mean age of 21), it is possible to consider some situations in agreement with Hershner *et al*. (2014) (5): “The circadian rhythm and homeostatic impulse are factors that “govern” sleep. The circadian rhythm (internal clock) helps regulate sleep cycles / wakefulness and the hormonal secretions, while the homeostatic impulse of sleep increases the necessity of sleep to the extent that the period of wakefulness increases. Physiologically, adolescents tend to have a late circadian preference and are “night owls”. This age group could have a smaller homeostatic sleep impulse, and consequently, are less sleepy at night. The typical adult circadian period is 24.1 hours, in comparison with the period of an adolescent of 24.27 hours: it is this longer period that makes it easier for the hour of sleep to be later. Therefore, by the fact that adolescents feel more wakefulness at night, they have more difficulty falling asleep, which leads them to sleep later at night. Consequently, they end up having insufficient sleep during the school week, making their recuperation of sleep occur on the weekend”. These situations are possibly associated with hormonal alterations which occurred in puberty (2,5). It is worth pointing out that puberty, which frequently occurs in conjunction with adolescence, is a biological phenomenon defined by a constellation of events (e.g., development of secondary sexual characteristics and modulation of muscles and fat), which are driven by increases in gonadal and adrenal hormones (30). Thus, based on the mean age obtained in this study, it is possible to suggest that there was a propensity for a quantity of insufficient hours of sleep among the evaluated students, and as a consequence, for EDS.

Beyond the intrinsic and physiologic factors (late circadian preference and smaller homeostatic sleep impulse) discussed prior, a reduction of sleep is also frequently attributed to extrinsic factors, such as artificial light, use of caffeine, lack of physical activity, absence of rules at the hour of sleep, and availability of communication and information technology (e.g., cellular phones, Tablets, computers, etc.) (2). In addition, sleep insufficiency could be related to social commitments and family dynamic and affection problems (2). Therefore, the intrinsic and extrinsic factors cited could have contributed to the high probability of EDS in the 33.6% of the students (Table 1). It is worth restating that from 18 to 25 years, the duration of sleep should be 7 to 9 hours (2).

A fundamental characteristic of depression is disturbed sleep, thus, insufficient sleep could increase the symptoms of depression (5). It is worth mentioning that prolonged latency of sleep could be associated to the loss of pleasure, feelings of punishment, and an aversion to oneself (5). Table 2, which corroborates this, shows a positive correlation between EDS and depression (Rank Correlation= 0.174; *p*= 0.049), therefore the higher the level of EDS, the greater the symptoms or levels of depression, and vice-versa. This corroborates the fact that depression is one of the causes of EDS (7).

In this study, the students with high probability of having EDS (33.6%) reported that they drove to the university, which is outside of the city, driving a car or motorcycle, and frequently gave a ride to other students. This situation could be worrisome, since according to Pagel (2009), people with EDS have a high risk of causing vehicular accidents (7). Because of this, it is recommended that odontology colleges develop campaigns in respect to the importance of healthy sleep.

Pagel (2009), also emphasized that EDS could cause work accidents (7). The odontology students evaluated with high probability of having EDS perform various types of interventions with patients (e.g., surgery, endodontic treatment, etc.) during the week. During these procedures the attention of the student is fundamental, since the student could be handling odontology materials such as scalpel blades, needles, etc. Therefore, a student with EDS during a clinical attendance could cause a penetrating accident (drill/cut) in the region of the head or neck of his/her patient, resulting in an uncomfortable situation between the student and patient, and possible judicial problems. In addition, due to a penetrating accident, a cross-contamination could even occur between the student and the patient (e.g., human immunodeficiency virus, hepatitis C, etc.).

All students that reported bruxism through the Pintado *et al*. (1997) questionnaire (24) reported both types of bruxism (sleep and nighttime). In a study performed in 2019, sleep bruxism was considered a strong predictor of wakefulness bruxism and vice-versa, when they are self-reported (31). Thus, reporting sleep bruxism significantly increases the chance of reporting daytime bruxism and vice-versa (29). This information (29,31) could explain the situation observed in the present study.

Daytime bruxism could be considered a response to stress and anxiety (1,31). In addition, daytime bruxism shows greater sensitivity to emotional alterations than sleep bruxism (31). This situation could explain the positive correlation of bruxism with anxiety (Rank Correlation= 0.255; *p*= 0.004) and stress (Rank Correlation= 0.201; *p*=0.023) (Table 2). Thus, possibly, daytime bruxism has greater prevalence in the present study. In addition, the fact that the bruxism evaluated is predominantly the daytime variety would also explain the fact that there was no correlation of EDS with bruxism (*p*=0.354) (Table 2). It is worth restating that EDS could originate as an outcome of micro-awakenings caused by sleep bruxism, which is caused by OSA (32). According to Martynowicz *et al*. (2019), one of the hypotheses that connects sleep bruxism with OSA is that the activity performed during nocturnal bruxism protects the individual against OSA; the way that the mandible is projected, restoring the permeability of the air passages (32), is resulting in a micro-awakening. Therefore, as participants with high probability of having OSA were excluded, this could also corroborate this absence of correlation between bruxism and EDS (*p*=0.354).

The onset of anxiety disorders peaks during adolescence and early adulthood, with women having a higher risk of acquiring this disorder compared to men (33,34). This corroborates the result of this study as the mean age was 21 years (adolescence period is between 12 and 25 years old) (30), and there was a positive correlation between females and anxiety (Rank correlation = 0.178; *p*= 0.045). In addition, there was a positive correlation of females with stress (Rank Correlation=0.199; *p*= 0.024). This could have occurred since women are more affected by stress factors than men, since they consider these stresses more threatening (34). Thus, as women stress more easily, this could cause a reduction in the level of estrogen, which could affect the sleep quality of women (20,35). This could explain the positive correlation between females and EDS (Rank Correlation= 0.209; *p*= 0.018) (Table 2). It is important to mention that it is not clear if testosterone affects the sleep of men (35).

A limitation of this study was that there was no verification of which type of bruxism (of sleep or daytime) had greater prevalence among the odontology students. In addition, transversal studies (such as is the case of the present study) are commonly used in epidemiologic studies to know the factors and associated risks, but not to evaluate the causes (1,10). Thus, longitudinal studies about the subject addressed in this article, with representative samples, must be performed (1,10).

## Conclusions

A high probability of EDS was present in 33.6% of odontology students, and EDS had a positive correlation with females and depression. The present study recommends awareness campaigns in odontology colleges about the importance of healthy sleep.
